# Ultra-processed foods and non-alcoholic fatty liver disease: an updated systematic review and dose–response meta-analysis

**DOI:** 10.3389/fnut.2025.1631975

**Published:** 2025-07-11

**Authors:** Jinghong Zhang, Long Shu, Xiaopei Chen

**Affiliations:** ^1^Department of Endocrinology, Zhejiang Hospital, Hangzhou, Zhejiang, China; ^2^Department of Clinical Nutrition, Zhejiang Hospital, Hangzhou, Zhejiang, China

**Keywords:** ultra-processed foods, non-alcoholic fatty liver disease, systematic review, dose–response, meta-analysis, nutritional epidemiology

## Abstract

**Background:**

Studies reported a significant association between ultra-processed food (UPF) consumption and increased risk of non-alcoholic fatty liver disease (NAFLD), but these have produced conflicting results. Thus, we conducted a systematic review and meta-analysis of existing observational studies to ascertain the association between UPF consumption and risk of NAFLD.

**Methods:**

We systematically searched PubMed, Web of Science, Embase and China National Knowledge Infrastructure (CNKI) without language restrictions for eligible studies published from database inception until 31 March 2025. Pooled relative risks (RRs) with 95% confidence intervals (CIs) were estimated using random-effects or fixed-effects models depending on heterogeneity.

**Results:**

Overall, 10 articles involving 513,440 participants and 20,637 NAFLD cases were included. Highest UPF consumption was associated with a 22% increased risk of NAFLD compared to the lowest consumption (RR = 1.22, 95%CI: 1.14–1.31, *p* < 0.001), with significant heterogeneity (*I*^2^ = 78.5%; *P*_heterogeneity_ < 0.001). A 10% increment in UPF consumption was associated with a 6% higher risk of NAFLD (RR = 1.06; 95%CI: 1.04–1.09, *I*^2^ = 75.9%; *p* < 0.001). Dose–response analysis showed a linear trend association between UPF consumption and risk of NAFLD (RR = 1.02; 95%CI: 0.98–1.07, *P_dose–response_ =* 0.295, *P_nonlinearity_ =* 0.541). Subgroup analyses revealed the positive associations between UPF consumption risk of NAFLD in the subgroups of 24-h dietary recalls (RR = 1.35, 95%CI: 1.24–1.46, *p* < 0.001), cross-sectional studies (RR = 1.29; 95%CI: 1.11–1.31, *p* = 0.001) and sample size<5,000 (RR = 1.20; 95%CI: 1.02–1.42, *p* = 0.030), with less evidence of heterogeneity.

**Conclusion:**

This study suggests that high UPF consumption is associated with an increased risk of NAFLD, albeit with substantial heterogeneity. Our findings underscore the importance of limiting UPF consumption in the prevention of NAFLD. Future studies with longitudinal designs are required to elucidate underlying mechanisms and confirm causality.

## Introduction

Non-alcoholic fatty liver disease (NAFLD), now also known as metabolic dysfunction-associated steatotic liver disease (MASLD), has emerged as the most common chronic liver disease worldwide, affecting approximately 30% of the global population (1, 2). A recent systematic review and meta-analysis reported a striking rise in global prevalence of NAFLD, now estimated at 32.4% among adults (3). In China, parallel with the ongoing obesity epidemic, NAFLD has become a serious health problem (4). Meanwhile, NAFLD is also a significant health burden in the United States, affecting more than 80 million people (5). These data reflect the urgency and necessity of implementing effective strategies to prevent NAFLD. The pathogenesis of NAFLD involves complex interactions among genetic predisposition, environmental factors, metabolism and gut microbiota (6). Therefore, identifying modifiable risk factors is essential for developing effective preventive strategies.

Extensive research over recent decades has firmly established diet as a critical factor in the development and progression of NAFLD (7). In particular, previous studies have primarily reported the associations between intakes of overall dietary patterns, individual foods, nutrients and risk of NAFLD (8–10). However, less attention has been paid to the impact of food processing degree on NAFLD. In 2009, the concept of NOVA food classification system was proposed by Brazilian researchers to enable categorization of all food and beverage items, according to the nature, extent and purpose of food processing (11). This system divided food and beverage items into four groups (unprocessed/minimally processed food, processed culinary ingredients, processed foods and ultra-processed foods). Of note, UPFs are typically ready-to-eat, highly palatable, cheap and characterized by high amounts of added sugars, saturated fat, *trans* fat, salt and high energy density, as well as low dietary fiber, vitamins and minerals (12). Over the last few decades, UPF consumption has drastically increased, accounting for 25% ~ 60% of daily energy intake worldwide (12, 13). In fact, the impact of UPF consumption on health recently has attracted considerable scientific interest. As yet, many observational studies have reported the associations between UPF consumption and multiple adverse outcomes, such as overweight/obesity, type 2 diabetes, cardiovascular disease and cancers (14–17). At present, only limited epidemiological studies have specifically reported the association between UPF consumption and NAFLD risk (18–27), and conclusions are not entirely consistent. Although a vast majority of studies have suggested a positive association between higher UPF consumption and increased risk of NAFLD (18–22, 24, 25, 27), other studies reported the null findings (23, 26). For example, Tianjin Chronic Low-grade Systemic Inflammation and Health (TCLSIH) cohort study by Zhang et al. found an increased risk of NAFLD with higher UPF consumption (22). In contrast, an Israeli cross-sectional study reported no significant association between high UPF consumption and risk of NAFLD (OR = 1.12; 95%CI: 0.78–1.59) (23). To our knowledge, only one meta-analysis by Henney et al. (28) has evaluated the association between UPF consumption and NAFLD risk. But, the aforementioned meta-analysis included limited studies, and had relatively small sample size as well as methodological limitation. For instance, this meta-analysis included the studies of Odegaard et al. (29) and Rahimi-Sakak et al. (30) which reported the association between specific food groups (e.g., fast food, and processed meat) and risk of NAFLD. Notably, in the last 2 years, several large cohort studies from United Kingdom and Korea have also been published (18, 20, 27). Therefore, to clarify the effect of UPF consumption on NAFLD risk, we conducted an updated systematic review and dose–response meta-analysis to incorporate available evidence of observational studies published from database inception to March 9, 2025.

## Methods

The current systematic review and meta-analysis adhered to the Preferred Reporting Items for Systematic Reviews and Meta-Analyses (PRISMA) guidelines (31). Additionally, because all included studies were observational in design, we followed the Meta-analyses of Observational Studies in Epidemiology (MOOSE) guidelines for meta-analysis of observational studies (32).

### Search strategy

A comprehensive literature search in PubMed, Web of Science, Embase and CNKI databases was conducted to retrieve relevant articles published from database inception up to 31 March 2025 (data last searched), with the following keywords or free-text terms: (“ultra-processed foods” OR “UPFs” OR “ultra-processed food” OR “UPF” OR “NOVA food classification”) AND (“non-alcoholic fatty liver disease” OR “NAFLD” OR “fatty liver disease” OR “metabolic dysfunction-associated fatty liver diseases” OR “MASLD”). Searches were limited to human studies without any restrictions regarding publication date and language. Furthermore, reference lists of eligible articles and prior systematic reviews of relevant topics were also reviewed to lessen the chance of missing potentially relevant articles. The literature search was conducted by two independent authors (X.-P. C and L. S.). The details of search strategy are described in the Supplementary Table S1.

### Study selection

Two authors (X.-P. C and J.-H. Z.) independently examined the titles and abstracts of all articles retrieved in the initial literature search to identify studies that reported the correlation between UPF consumption and risk of NAFLD. To be included in this updated systematic review and meta-analysis, the following criteria had to be met: (1) observational studies, including cohort, case–control, nest case–control, case-cohort or cross-sectional studies, performed in adults (≥18 years); (2) considered UPF consumption as the exposure variable; (3) UPF was defined based upon the NOVA food classification; (4) considered NAFLD as the outcome variable; (5) studies reporting the association between UPF consumption and risk of NAFLD, providing the estimates of relative risks (RRs), hazard ratios (HRs), odds ratios (ORs) along with their corresponding 95% confidence intervals (CIs) (or sufficient data to calculate them); (6) If the original data in the retrieved studies lacked sufficient detail, the corresponding author will be contacted by email. Additionally, studies were excluded if they met one of the following criteria: (1) animal, cell culture, and *in vitro* studies; (2) non-observational studies, including congress abstracts, reviews, editorials, case reports, book chapters, and letters; (3) did not use the NOVA food classification system to define UPF (assessed the only specific food or food groups, such as sugar-sweetened drinks, processed meat); (4) Secondary steatosis, e.g., fatty liver disease, non-alcoholic steatohepatitis, viral hepatitis; (5) did not provide the HRs, RRs or ORs with corresponding 95%CIs; (6) Irrelevant articles; (7) studies performed in the pediatric participants (aged < 18 years). The PECOS criteria for this meta-analysis is described in Table 1.

**Table 1:** The PECOS criteria used for this meta-analysis.

Population	Adults
Exposure	Ultra-processed food consumption
Comparison	Highest versus lowest categories of exposure and each 10% increment in exposure
Outcomes	Non-alcoholic fatty liver disease
Study design	Cohort, case–control or cross-sectional studies

PECOS, participant, exposure, comparison, outcome, and study design.

### Data extraction and quality assessment

For each eligible article, we used a predefined form to extract the required information on first author’s name, year of publication, study design, study region, sample size, number of total participants and NAFLD cases, mean age/age range, follow-up duration for cohort studies, dietary assessment method, and confounding variables that were adjusted for in the statistical analysis. In this study, two authors (J.-H. Z. and L. S.) independently utilized the Newcastle-Ottawa Scale (NOS) to judge the methodological quality of all included studies. The NOS consisted of three domains: selection of participants, comparability of participants, and ascertainment of outcome/exposure of interest, which was designed for non-randomized studies in meta-analyses (33). The total NOS score ranges from 0 to 9 points, with higher scores indicating higher study quality. Studies with total NOS scores ≥7 points were judged as high quality (34). Any discrepancies arising during data extraction and quality assessment were resolved via joint discussion and/or consultation with the corresponding author (X.-P. C).

### Ascertainment of UPF

According to the NOVA classification, all food and beverage items were divided into four different groups, including unprocessed or minimally processed foods, processed culinary ingredients, processed foods and UPFs (11). Existing literature indicates that UPFs are typically ready-to-eat, hyper-palatable, cheap and characterized by high energy density, high intake of added sugars, salt, saturated and *trans*-fats, as well as low consumption of dietary fiber, vitamins and minerals (12). Typical examples of UPF include biscuits and cakes, crisps, sugar-sweetened beverages, pizza, processed meats, desserts, etc.

### Data synthesis and analysis

The RRs and their 95%CIs were considered as the effect estimate in this updated systematic review and meta-analysis. Meanwhile, we assumed that the HRs were approximately equal to RRs (35). For cross-sectional or case–control studies, ORs were converted to RRs by the following formula: RR = OR/[(1-P_0_) + (P_0_*OR)], where P_0_ shows the incidence of NAFLD in the non-exposed group (36). The pooled RRs and 95%CIs of NAFLD were calculated by comparing the highest and the lowest categories of UPF consumption and for each 10% increment in UPF consumption. The Cochran’s *Q* test and *I*-squared (*I*^2^) statistic were used to evaluate between-studies heterogeneity with estimates associated with *p*-values of Cochran’s Q test <0.05 and *I^2^* ≥ 50% considered to be substantial heterogeneity (37). If there was substantial heterogeneity between studies, RRs were calculated using a random-effects meta-analysis with the DerSimonian and Laird method. Otherwise, fixed-effects model was used to pool the RRs (38). Given the expected heterogeneity of the included studies, leave-one-out sensitivity and subgroup analyses were used to detect the potential sources of heterogeneity. Subgroup analyses were performed based on study design (cohort or cross-sectional studies), dietary assessment method (FFQ or 24-h dietary recall), study region (Western countries or other countries), sample size (≥5,000 or <5,000), study quality (≥7 or <7), and mean age (≥50 or <50). Sensitivity analysis was conducted to confirm whether the pooled RRs were robust or sensitive to the impact of a certain study. Univariable meta-regression analyses were also performed to test the effect of specific variables (i.e., sample size, study area, and dietary assessment methods) on the effect size for the association between UPF consumption and NAFLD. Publication bias was assessed via the visual inspection of funnel plots and quantified by Begg’s test and Egger’s regression asymmetry test (39). If the results indicated evidence of publication bias, the trim-and-fill method was used to re-calculate the results (40). Finally, we also performed a dose–response meta-analysis to estimate the trend from the correlated log RRs across the categories of UPF consumption. A two-stage GLST model based on generalized least squares was used to examine potential linear or non-linear dose–response association between UPF consumption and risk of NAFLD. We used UPF consumption modeling and restricted cubic splines with three knots at fixed percentiles (10, 50, and 90%) of the distribution. All statistical analyses were carried out using STATA, version 17.0 (StataCorp, College Station, TX, United States), and a two-sided *p-*value of ≤0.05 was regarded as statistically significant.

## Results

### Overview of included studies for this updated systematic review and meta-analysis

**Figure 1:**
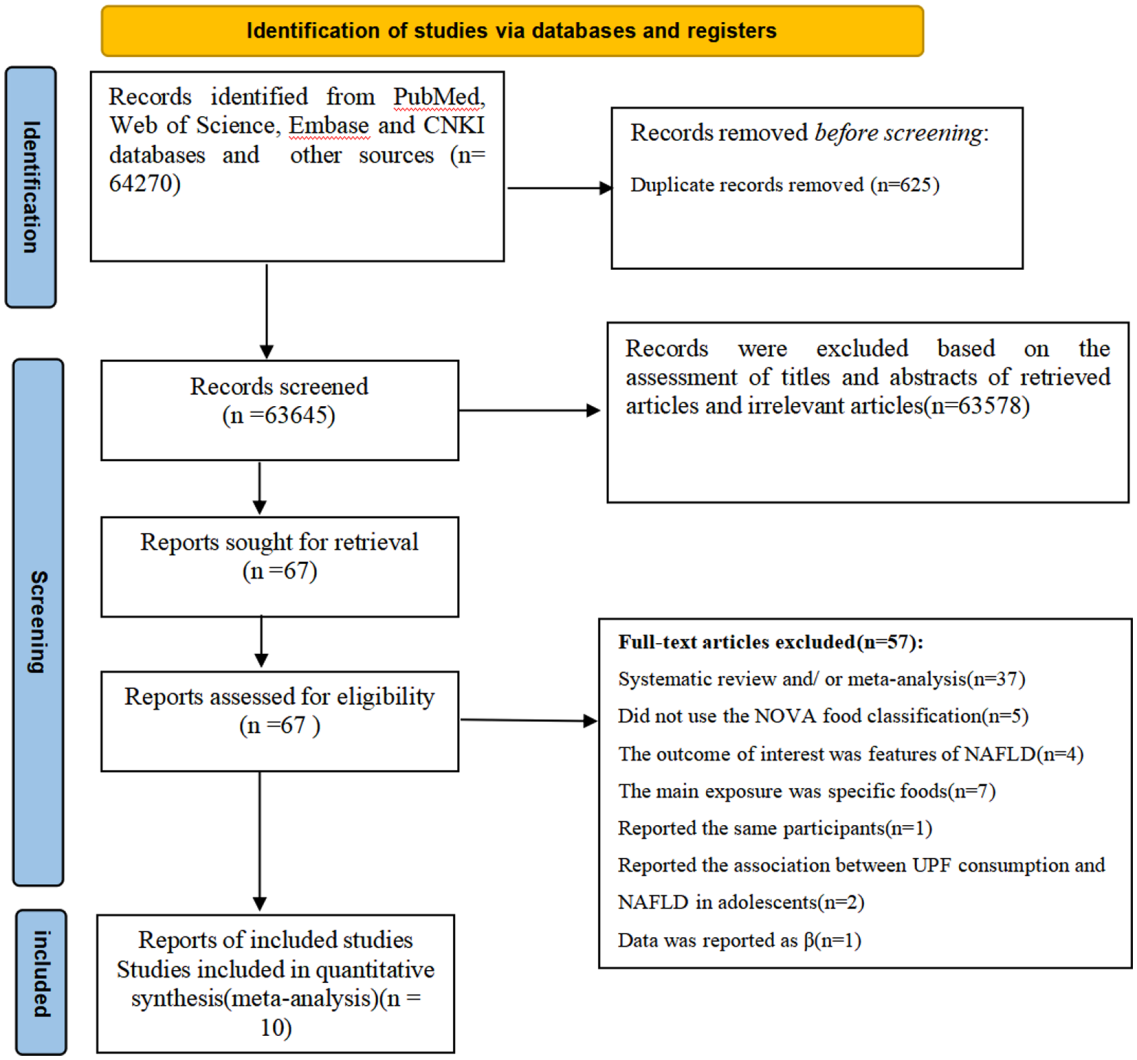
Flowchart of the study selection process for this systematic review and meta-analysis.

Figure 1 summarized the results of the literature search and study selection. During the initial literature screening, we identified 64,270 potentially relevant articles through four databases and other sources. After removing 625 duplicates, 63,645 records were preliminarily screened. Subsequently, 63,578 articles were excluded based on the examining of titles and abstracts of retrieved articles and irrelevant articles. The remaining 67 full-text articles were reviewed in details by two independent authors (X. P. C and L. S.) and 57 articles were excluded for the following reasons: systematic review and/or meta-analysis (*n* = 37), did not use the NOVA food classification (*n* = 5), report the association between UPF consumption and risk of NAFLD in adolescents (*n* = 2), the outcome of interest was features of NAFLD (*n* = 4), the main exposure was specific foods, such as sugar-sweetened beverages, desserts, and processed meats (*n* = 7), reported data as *β* coefficient (*n* = 1), and reported the same participants (*n* = 1). Ultimately, a total of 10 articles fulfilled the inclusion criteria and were included in the final analysis.

### Characteristics of eligible studies

The main characteristics of these studies included in the meta-analysis are presented in Table 2. A total of 10 articles (involving 513,440 participants and 20,637 NAFLD cases), published between 2021 and 2025, were included in the final analyses. All the eligible studies had an observational design. Six of all included studies were cohort studies (18, 20, 22, 24, 25, 27), and the remaining four were cross-sectional studies (19, 21, 23, 26). Two of included studies were from United Kingdom (18, 20), two from United States (19, 21), two from Israel (23, 26), one from China (22), one from Brazil (25), one from Korea (27), and one from Spain (24). The follow-up duration of cohort studies ranged from 4.2 to 10.5 years. Sample size of included studies ranged from 286 to 173,889. The age of participants across studies ranged from ages 20 to above. Five studies used fatty liver index for the diagnosis of NAFLD (19, 24–27), two studies used ultrasonography (22, 23), two used hospital records (18, 20) and one study used the controlled attenuation parameter (21). UPF consumption was self-reported through food frequency questionnaires (FFQs) and 24-h dietary recalls across all published studies. Specifically, six articles used an FFQ (22–27), and the remaining four studies used 24-h dietary recalls (18–21). All the included studies classified UPF consumption basing on the NOVA food classification systems (18–27). As reported on Table 3, eight out of all included studies received NOS scores ≥7 points, and were classified as of high-quality (18–22, 24, 25, 27). Additionally, the remaining two studies were classified as of medium-quality (23, 26).

**Table 2:** The main characteristics of these studies included in this systematic review and meta-analysis.

Author Publication Year	Study region	Study design	Total number of participants	Age	Diagnostic tool for NAFLD	Exposure assessment	Adjustment or matched for in analyses	Outcomes
Zhang et al. (2024) (18)	United kingdom	Cohort	143,073 (1,445 cases)	40-69y	Hospital records	24 h dietary records	Age (time scale), stratification by sex and ethnicity, exposure variable only, index of multiple deprivation and education, BMI continuous at baseline, smoking status (current/never/previous), alcohol consumption (never/<3 times per week/3 times per week), and physical activity level (low/moderate/high).	Highest quartile 4 vs. lowest quartile 1 (HR = 1.26,95%CI:1.11–1.43); Per 10% absolute increment in ultra-processed food intake (HR = 1.09, 95%CI: 1.06–1.13)
Liu et al. (2023) (19)	United States	Cross-sectional	6,545 (2,224 cases)	≥20y	Fatty liver index	24 h dietary records	Age, gender, race/ethnicity, education level, family income to poverty ratio, marital status, smoking status, BMI, biochemistry factors (log-transformed): serum ALT, fasting TAG, total cholesterol and uric acid, dietary pattern (total HEI score), added sugar, saturated fat, refined grains.	Highest quartile 4 vs. lowest quartile 1 (OR = 1.78,95%CI: 1.29–2.15); 10% increase in the dietary contribution of UPF (OR = 1.14, 95%CI: 1.07–1.20)
Zhao et al. (2024) (20)	United kingdom	Cohort	173,889 (1,108cases)	40–69y	Hospital records	24 h dietary records	Age, sex, ethnicity, Townsend deprivation index, smoking status, alcohol drinking, physical activity, body mass index, aspirin use, self-reported diabetes, and total energy intake	Highest quartile 4 vs. lowest quartile 1 (HR = 1.43,95%CI: 1.21–1.70); Per 100 g increment in ultra-processed food intake (HR = 1.04, 95% CI: 1.02–1.05)
Zhao et al. (2023) (21)	United States	Cross-sectional	2,734 (1,053 cases)	≥20y	Controlled attenuation parameter	24 h dietary records	Age, sex, race/ethnicity, education, smoking pack-years, alcohol drinking, physical activity (adults only), and total energy intake	Highest quintile 5 vs. lowest quintile 1 (OR = 1.72,95%CI: 1.01–2.93); Per 100 g increment in ultra-processed food intake (OR = 1.02, 95% CI: 0.99–1.07)
Zhang et al. (2022) (22)	China	Cohort	16,168 (3,752 cases)	18–90y	Ultrasonography	FFQ	Age, sex, body mass index, smoking status, alcohol drinking status, educational level, occupation, monthly household income, physical activity, family history of disease (including cardiovascular disease, hypertension, hyperlipidaemia and diabetes), depressive symptoms, total energy intake, healthy diet score, hypertension, hyperlipidaemia and diabetes.	Highest quartile 4 vs. lowest quartile 1 (OR = 1.18,95%CI: 1.07–1.30); Per SD increment in ultra-processed food intake (HR = 1.06, 95%CI: 1.03–1.09)
Ivancovsky-Wajcman et al. (2021) (23)	Israel	Cross-sectional	786 (305 cases)	40-70y	Ultrasonography	FFQ	Age, gender, BMI, saturate fatty acids and protein intake (% of total kcal), physical activity (hours/week), coffee (cups/day) and fiber intake (gr/day).	Highest vs. lowest categories of UPF consumption (OR = 1.12, 95%CI: 0.78–1.59).
Konieczna et al. (2022) (24)	Spain	Cohort	5,867 (4,934 cases)	55–75y	Fatty liver index	FFQ	Age at inclusion, sex, study arm, follow-up time (months), baseline educational level, smoking habits, height, as well as repeatedly measured at baseline and every6 months thereafter physical activity, sedentary behavior, and alcohol intake (all continuous).	Highest quintile 5 vs. lowest quintile 1 (OR = 3.73, 95%CI: 3.10–4.35); Per 10% increment in ultra-processed food intake (OR = 1.60, 95% CI: 1.24–1.96).
Canhada et al. (2024) (25)	Brazil	Cohort	13,316 (1,893 cases)	35–74y	Fatty liver index	FFQ	Age, sex, race/color, school achievement, per capita family income, smoking, physical activity, and alcohol consumption.	Highest vs. lowest categories of UPF consumption (HR = 1.12, 95%CI: 1.08–1.16).
Fridén et al. (2022) (26)	Israel	Cross-sectional	286 (66 cases)	40–70y	Fatty liver index	FFQ	Sex, education level, physical activity level, smoking status, dietary factors and BMI.	Highest tertile 3 vs. lowest tertile 1 (RR = 1.30, 95%CI: 0.67–2.56)
Fu et al. (2025) (27)	Korea	Cohort	44,642 (1,562 cases)	40–69y	Fatty liver index	FFQ	Age, total energy intake, the mean intake of processed foods, unprocessed or minimally processed foods, and processed culinary ingredients, income level, education level, physical level, drinking status, smoking status, sleep duration, stress status, prevalence of obesity, diabetes, hypertension, and dyslipidemia at baseline.	Men: highest quartile 4 vs. lowest quartile 1 (HR = 1.35, 95%CI: 1.06–1.71); Per SD increment in UPF intake (HR = 1.10, 95%CI: 1.02–1.18). Women: highest quartile 4 vs. lowest quartile 1 (HR = 1.48, 95%CI: 1.19–1.86); Per SD increment in UPF intake (HR = 1.09, 95%CI: 1.02–1.16).

BMI, body mass index; CI, confidence interval; FFQ, food frequency questionnaire; HR, Hazard ratio; NAFLD, non-alcoholic fatty liver disease; OR, odds ratio; UPF, ultra-processed food.

**Table 3:** Ultra-processed food consumption and risk of non-alcoholic fatty liver disease: assessment of study quality.

Studies	Selection				Comparability		Outcome			Score
	1	2	3	4	5A	5B	6	7	8	
Cohort										
Zhang et al. (2024) (18)	*	*	*	*	*	*	*	*	*	9
Zhao et al. (2024) (20)	*	*	*	*	*	*	*	*	*	9
Zhang et al. (2022) (22)	*	*	*	*	*		*	*	*	8
Konieczna et al. (2022) (24)	*	*	*	*	*		*	*	*	8
Canhada et al. (2024) (25)	*	*	*	*	*		*	*	*	8
Fu et al. (2025) (27)	*	*	*		*	*	*	*	*	8
Cross-sectional										
Liu et al. (2023) (19)	*	*	*		*		*	*	*	7
Zhao et al. (2023) (21)	*	*	*		*		*	*	*	7
Ivancovsky-Wajcman et al. (2021) (23)	*	*	*		*		*	*		6
Fridén et al. (2022) (26)	*	*	*		*		*	*		6

^*^For case–control studies, 1 indicates cases independently validated; 2, cases are representative of population; 3, community controls; 4, controls have no history of NAFLD; 5A, study controls for the most important factor; 5B, study controls for additional factor(s),e.g. cigarette smoking body mass index, total energy intake; 6, ascertainment of exposure by blinded interview or record; 7, same method of ascertainment used for cases and controls; and 8, non-response rate the same for cases and controls. For cohort studies, 1 indicates exposed cohort truly representative; 2, non-exposed cohort drawn from the same community; 3, ascertainment of exposure by secure record (e.g. surgical records) or structured interview; 4, outcome of interest was not present at start of study; 5A, study controls for the most important factor; 5B, study controls for additional factor(s); 6, assessment of outcome is based on independent blind assessment or record linkage; 7, follow-up long enough (≥5 years) for outcomes to occur; and 8, adequacy of follow up of cohorts (all participants complete follow up or >90% participants complete follow up).

### UPF consumption and risk of NAFLD

**Figure 2:**
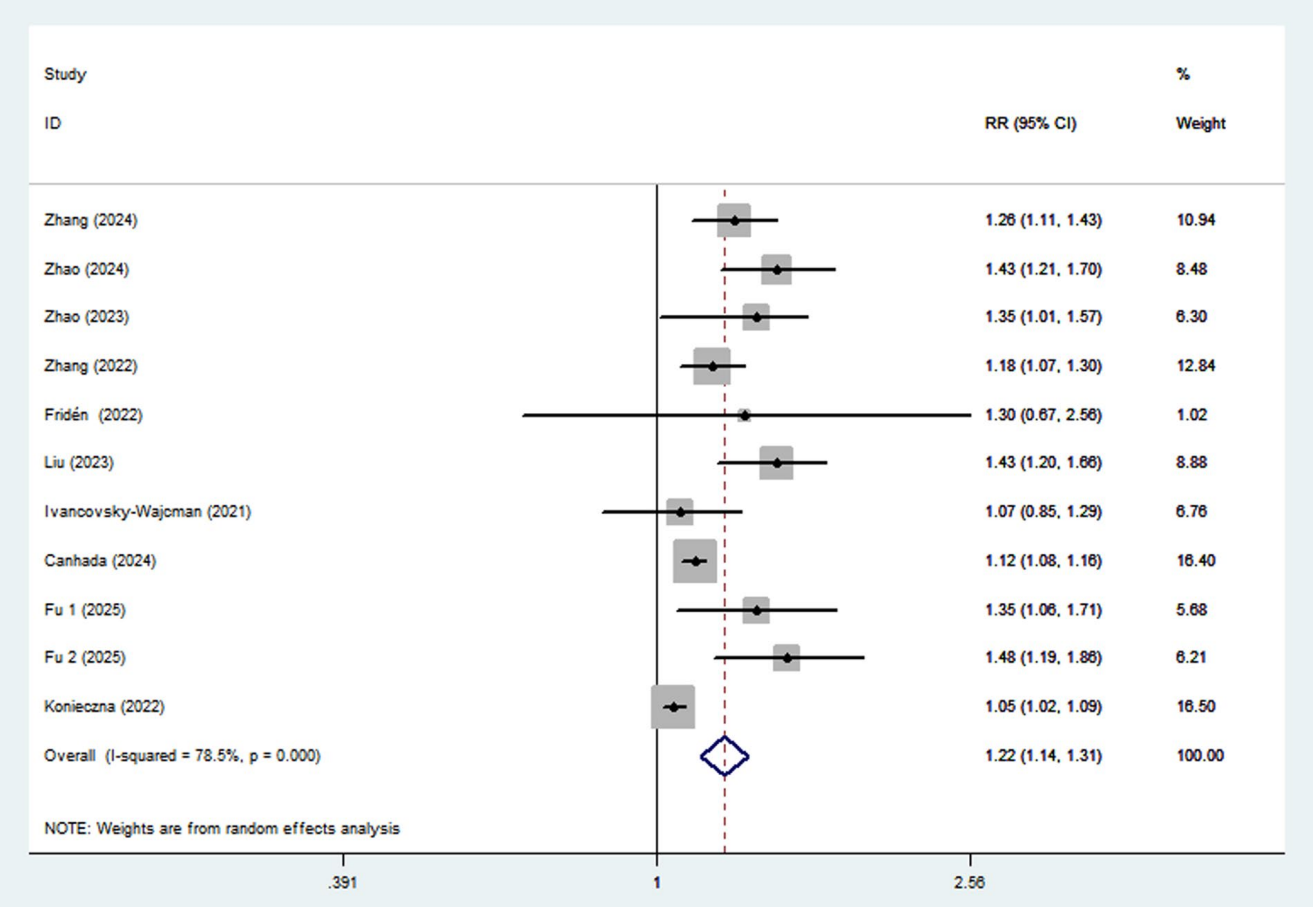
Forest plot of the association between UPF consumption and NAFLD risk.

**Figure 3:**
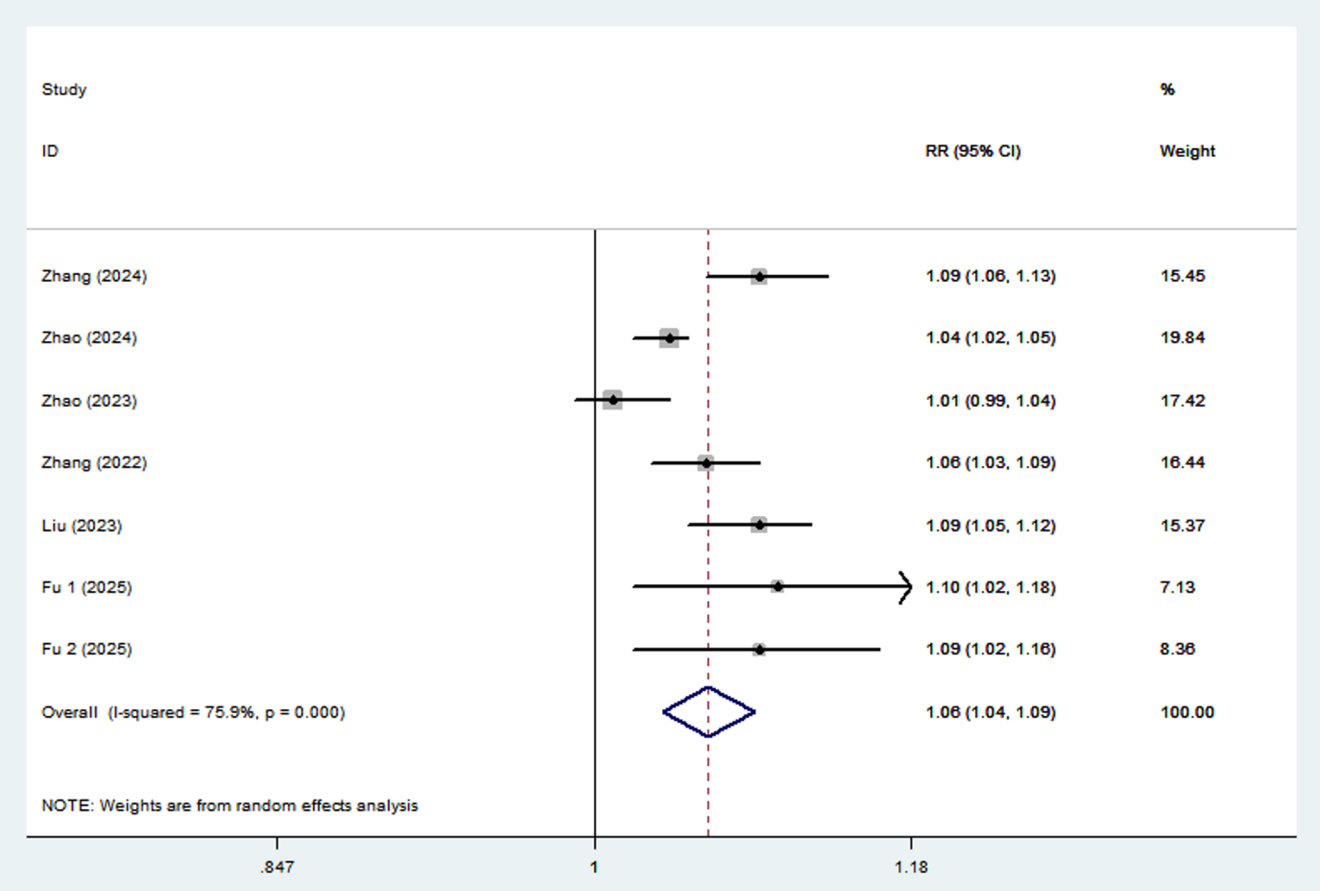
Forest plot of the association between each 10% increment in UPF consumption and NAFLD risk.

Ten articles involving a total of 513,440 participants and 20,637 NAFLD cases, were included in this meta-analysis. Highest UPF consumption was associated with a 22% increased risk of NAFLD compared to the lowest consumption (RR = 1.22, 95%CI: 1.14–1.31, *p* < 0.001) (Figure 2), with significant heterogeneity (*I*^2^ = 78.5%; *P*_heterogeneity_ < 0.001). A 10% increment in UPF consumption was associated with a 6% higher risk of NAFLD (RR = 1.06; 95%CI: 1.04–1.09, *I*^2^ = 75.9%; *p* < 0.001) (Figure 3).

### Dose–response analysis

**Figure 4:**
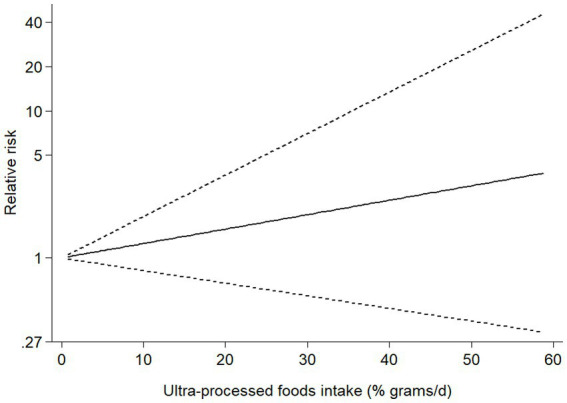
Dose-response analysis for the association between UPF consumption and NAFLD risk.

Six studies (4 cohort and 2 case–control studies) were included in the dose–response analysis for the association between UPF consumption and risk of NAFLD (Figure 4). The results showed a linear trend association between UPF consumption and risk of NAFLD (RR = 1.02; 95%CI: 0.98–1.07, *P_dose–response_ =* 0.295, *P_nonlinearity_ =* 0.541).

### Subgroup analyses and meta-regressions

Given the substantial heterogeneity in this meta-analysis (*I*^2^ = 78.5%, *p <* 0.001), subgroup analyses were undertaken to further explore the potential sources of heterogeneity across studies (Table 4). In our analyses, subgroup analyses were stratified based on study design (cohort or cross-sectional studies), dietary assessment method (FFQ or 24-h dietary recalls), study region (Western countries or other countries), sample size (≥5,000 or <5,000), study quality (≥7 or <7), and mean age (≥50 or <50). The results of subgroup analyses suggested that high UPF consumption was significantly associated with an increased risk of NAFLD in the subgroups of 24-h dietary recalls (RR = 1.35, 95%CI: 1.24–1.46, *p* < 0.001), and no heterogeneity was found (*p =* 0.559; *I*^2^ = 0.0%). When analyzed separately by study design, the results showed a positive association between UPF consumption and NAFLD risk in cross-sectional studies (RR = 1.29; 95%CI: 1.11–1.31, *p* = 0.001). There was less evidence of heterogeneity between studies (*p* = 0.188; *I*^2^ = 37.3%). For sample size, we observed a positive association between UPF consumption and NAFLD risk in studies with sample size <5,000 (RR = 1.20; 95%CI: 1.02–1.42, *p* = 0.030), and there was less heterogeneity (*p =* 0.315; *I*^2^ = 13.5%). Meanwhile, a positive association between UPF consumption and risk of NALFD was also observed in the subgroups of age < 50 (RR = 1.29; 95%CI: 1.13–1.48, *p <* 0.001), and heterogeneity decreased to 55.0%. As shown in Supplementary Table S2, the results of univariable meta-regression analyses to explore the effects of potential moderator variables revealed no significant effects of study region and sample size on the association between UPF consumption and risk of NAFLD (*p* > 0.05).

**Table 4:** Subgroup analyses between UPF consumption and risk of NAFLD.

Study characteristic	Category	No. of studies	RR (95%CI)	*P*-values	Heterogeneity		
					*P*-values for within groups	*I*^2^ (%)	*P*-values for between groups
Overall		10	1.22 (1.14–1.31)	<0.001	0.000	78.5	
Study design	Cross-sectional	4	1.29 (1.11–1.31)	0.001	0.188	37.3	0.004
	Cohort	6	1.20 (1.12–1.29)	<0.001	0.000	82.1	
Dietary assessment method	FFQ	6	1.14 (1.07–1.22)	<0.001	0.000	70.2	<0.001
	24-h dietary recall	4	1.35 (1.24–1.46)	<0.001	0.559	0.0	
Study region	Western countries	6	1.25 (1.08–1.43)	0.002	0.000	85.3	0.053
	Other countries	4	1.21 (1.10–1.32)	<0.001	0.068	54.2	
Sample size	≥5,000	7	1.23 (1.14–1.33)	<0.001	0.000	83.8	0.307
	<5,000	3	1.20 (1.02–1.42)	0.030	0.315	13.5	
Study quality	≥7	8	1.24 (1.15–1.33)	<0.001	0.000	82.7	0.839
	<7	2	1.09 (0.89–1.33)	0.404	0.587	0.0	
Mean age	≥50	7	1.19 (1.11–1.29)	<0.001	0.000	78.1	0.001
	<50	3	1.29 (1.13–1.48)	<0.001	0.108	55.0	

CI, confidence interval; FFQ, food frequency questionnaire; NAFLD, non-alcoholic fatty liver disease; RR, relative risk; UPF, ultra-processed food.

### Publication bias and sensitivity analysis

As shown in Supplementary Figure S1, an examination of the funnel plot revealed little evidence of asymmetry. Similarly, Begg’s and Egger’s tests did not show the presence of significant publication bias (highest versus lowest categories of UPF consumption: Begg’s test: *p* = 0.640; Egger’s test: *p* = 0.108). Additionally, the association between UPF consumption and NAFLD risk might be affected by single study based on the results of sensitivity analysis. As shown in Supplementary Figure S2, the study by Konieczna et al. (24) exceeded the range and might be a potential source of heterogeneity. After excluding the studies of Konieczna et al. (24) and Canhada et al. (25) in the repeat analyses (Supplementary Figure S3), sensitivity analysis revealed a slight increase in the pooled RRs on the association between UPF consumption and risk of NAFLD (RR = 1.29; 95%CI: 1.21–1.39, *p* < 0.0001). Moreover, the heterogeneity also decreased from 78.5 to 27.5%.

## Discussion

To our knowledge, this is the most comprehensive systematic review and dose-response meta-analysis assessing the association between UPF consumption and risk of NAFLD. Our updated meta-analysis of 10 observational studies demonstrated that high UPF consumption was associated with a 22% higher risk of NAFLD. A 10% increment in UPF consumption was associated with a 6% higher risk of NAFLD. Dose–response analysis revealed a linear trend. Subgroup analyses revealed the positive association between high UPF consumption and NAFLD risk was more robust in the subgroups of cross-sectional studies, 24-h dietary recalls, mean age<50y, and sample size <5,000. While sensitivity analysis showed a slight increase in the pooled RRs when excluding the studies by Konieczna et al. (24) and Canhada et al. (25) in the repeat analysis, substantial heterogeneity warrants cautions interpretation of our findings. Overall, our findings strengthen the existing evidence linking high UPF consumption to NAFLD risk, and support the emphasis on the importance of reducing UPF consumption in the prevention of NAFLD.

In parallel with the global epidemic of obesity and type 2 diabetes, NAFLD prevalence has surged dramatically (41), affecting approximately 32.4% of adults worldwide (3). This alarming trend underscores the urgent need to identify modifiable risk factors, with dietary factors being consistently recognized as a key and modifiable risk factor for NAFLD (7). Over the past few decades, while numerous epidemiological studies have examined the associations between intakes of individual foods, nutrients or overall dietary patterns and risk of NAFLD (8, 9), research on UPF consumption and NAFLD remains limited. Until now, most studies have revealed a positive association between UPF consumption and NAFLD risk (18–22), but two Israeli cross-sectional studies reported null findings (23, 26). Very recently, in the Health Examinees (HEXA) study involving 44,642 participants aged 40–69 years, from the Korean Genome and Epidemiology Study, Fu et al. found that higher UPF consumption was linked to an increased risk of NAFLD (27). Similar to previous findings, our meta-analysis also found that high UPF consumption was associated with a higher risk of NAFLD (RR = 1.22, 95%CI: 1.14–1.31, *p* < 0.001). The reasons for discrepancies among studies are difficult to fully elucidate. But, methodological variations in UPF assessment, differences in UPF types and quantities consumed across diverse populations, and duration of study follow-up might explain part of these discrepant results (42). Therefore, to identify the effect of UPF consumption on NAFLD risk, we conducted a updated systematic review and dose–response meta-analysis.

Although the evidence regarding the positive association between UPF consumption and NAFLD risk remains inconsistent, several possible mechanisms have been proposed to explain this adverse effect. First, UPFs are generally regarded as high-energy density foods, containing a large amount of added sugars, salt, total fat and saturated fat, and having low content of dietary fiber and vitamins (43). Observational studies have shown that high sugar and salt intake is associated with an increased risk of NAFLD (44, 45). Additionally, insufficient intake of dietary fiber is associated with higher risk of obesity and insulin resistance (46), both of which are known risk factors for NAFLD. Second, UPFs often contain additives, such as carrageenan, which has been shown to impair insulin signaling and promote insulin resistance, a key driver of NAFLD (47). Artificial sweeteners and other additives may further exacerbate metabolic risk factors, including obesity and type 2 diabetes, which are closely associated with NAFLD (48). Third, packaging materials may introduce endocrine-disrupting compounds (e.g., phthalate and bisphenol A) into UPFs. Experimental evidence has indicated that these chemicals may contribute to insulin resistance, thereby elevating NAFLD risk (49). Fourth, high-temperature food processing may generate harmful compounds such as advanced glycation end products (AGEs) and acrylamide. Preclinical studies demonstrated that dietary AGEs promote NAFLD onset and progression (50). Additionally, previous studies also showed that acrylamide was positively associated with NAFLD in the United States population (51). Fifth, over-consumption of UPF alters gut microbiota composition, favoring pro-inflammatory microbial profiles. This dysbiosis may further aggravate NAFLD pathogenesis via gut-liver axis signaling (52, 53). Finally, the harmful effect of high UPF consumption on NAFLD may partly be attributed to low consumption of healthy foods, e.g., vegetables, fruits, and whole grains. It is well-known that these foods are rich in dietary fiber. As already aforementioned, dietary fiber is closely associated with the risk of obesity and insulin resistance (46), which are important risk factors for NAFLD. Taken altogether, these mechanisms may explain the positive correlation between high UPF consumption and risk of NAFLD.

Although our findings showed a positive association between high UPF consumption and NAFLD risk, substantial heterogeneity was observed in the included studies (*I*^2^ = 78.5%; *P*_heterogeneity_ < 0.001). Thus, subgroup analyses were performed to explore sources of heterogeneity basing on the study design (cohort or cross-sectional studies), dietary assessment method (FFQ or 24-h dietary recalls), study region (Western countries or other countries), sample size (≥5,000 or <5,000), study quality (≥7 or <7), and mean age (≥50 or <50). The results suggested that heterogeneity might be partly due to the differences in study design, dietary assessment method, sample size and mean age. Specifically, when dietary assessment method was 24-h dietary recall, the heterogeneity decreased from 78.5 to 0.0%. There are several possible explanations for substantial heterogeneity. First, four included studies used cross-sectional designs, inherently limiting causal inference for the observed association between UPF consumption and NAFLD risk. These findings may be further influenced by recall bias from dietary assessment methods (e.g., FFQs and 24-h dietary recall). Second, three of included studies had a relatively small sample size (<5,000 participants), potentially affecting statistical power and contributing to heterogeneity. Third, despite the RRs or ORs were all from the highest category (taking the lowest category as the reference), different studies divided the UPF score range into different intervals. It might introduce significant methodological heterogeneity. Fourth, the included populations come from seven countries, including United Kingdom, United States, Israel, Spain, Korea, China and Brazil with distinct dietary habits, which might explain the observed heterogeneity. Fifth, inconsistent adjustment for potential confounders across studies may contribute to the observed heterogeneity. Finally, significant heterogeneity persisted in subgroup analyses, indicating the presence of additional unknown confounding factors.

### Strengths and limitations

This systematic review and meta-analysis has several important strengths. First, as discussed previously, this systematic review and updated meta-analysis is the largest study to date that comprehensively assesses the association between UPF consumption and NAFLD risk. Compared with the aforementioned meta-analysis (28), we not only provided timely updates, but also included more participants and NAFLD cases, thereby enhancing the statistical power to determine a more reliable estimate of the association between UPF consumption and risk of NAFLD. Furthermore, our findings add to the existing evidence and support the emphasis on the importance of reducing UPF consumption in the prevention of NAFLD. Second, visual inspection of the funnel plots and statistical tests (e.g., Begg’s and Egger’s tests) did not indicate any significant publication bias. Third, we strictly screened the articles according to the predetermined inclusion and exclusion criteria. Fourth, all included studies adopted the NOVA classification system to define UPF, avoiding misclassification errors. Finally, despite substantial heterogeneity, we undertook subgroup and sensitivity analyses to explore potential sources of heterogeneity. In addition, dose–response analysis was conducted to further strengthen the association between UPF consumption and risk of NAFLD. Notwithstanding the aforementioned strengths, our study also has some limitations that should be mentioned. First, the observational design of the included studies does not allow for the establishing a causal association between high UPF consumption and the risk of NAFLD. Therefore, further prospective cohort studies or intervention trials are needed to confirm the association between UPF consumption and NAFLD risk. Second, most of the included studies used FFQs to gather dietary information, which may led to an under-or over-estimation of UPF consumption. Additionally, FFQs and 24-h dietary recalls were not explicitly designed to capture the degree of processing, and not validated to precisely measure UPF consumption based on the NOVA food classification. Third, even with some potential confounding variables adjusted, the possibility of residual confounding by certain unmeasured factors could not be excluded. Additionally, our analysis did not search gray literature extensively, which may introduce potential selective reporting bias. Fourth, significant heterogeneity (*I*^2^ = 78.5%) was observed in this meta-analysis. Despite conducting subgroup and sensitivity analyses to explore potential sources of heterogeneity, we were unable to fully account for the sources of inter-study heterogeneity. Finally, eight of the included studies originated from developed countries and two studies from developing countries, which might limit the generalizability of our findings.

## Conclusion

In conclusion, this updated systematic review and meta-analysis provides compelling evidence that higher UPF consumption is associated with an increased risk of NAFLD. Our findings added further evidence for the adverse effect of high UPF consumption on NAFLD, highlighting the importance of reducing UPF consumption in preventing NAFLD. Further research should prioritize large-scale prospective studies across diverse populations to validate these findings.

## Data Availability

The original contributions presented in the study are included in the article/Supplementary material, further inquiries can be directed to the corresponding author.
