# Supplementary material for: Ultra-processed foods and non-alcoholic fatty liver disease: an updated systematic review and dose–response meta-analysis
Source: Front Nutr. 2025 Jul 11;12:1631975. doi: 10.3389/fnut.2025.1631975 (PMC12289574; doi:10.3389/fnut.2025.1631975)
Supplement: Supplementary file 1 [file Table_1.doc]

**Search terms in PubMed, Embase and Web of Science**
(“ultra-processed foods”[all fields] OR “UPFs” [all fields] OR “ultraprocessed food” [all fields] OR “UPF” [all fields] OR “NOVA food classification” [all fields]) AND (“non-alcoholic fatty liver disease” [all fields] OR “NAFLD” [all fields] OR “fatty liver disease”[all fields] OR “metabolic dysfunction-associated fatty liver diseases”[all fields] OR “MAFLD”[all fields])

**Search terms in China National Knowledge Infrastructure (CNKI)**

(饮食 OR 营养) and 非酒精性脂肪肝

**Search terms in other sources**

The reference lists from included articles and prior reviews were also examined to find potentially relevant articles.
